# Dying Too Soon: Excess Mortality in Severe Mental Illness

**DOI:** 10.3389/fpsyt.2019.00855

**Published:** 2019-12-06

**Authors:** Liselotte D. de Mooij, Martijn Kikkert, Jan Theunissen, Aartjan T.F. Beekman, Lieuwe de Haan, Pim W.R.A. Duurkoop, Henricus L. Van, Jack J.M. Dekker

**Affiliations:** ^1^Department of Research, Arkin Mental Health Institute, Amsterdam, Netherlands; ^2^Department of Research, GGZ inGeest Mental Health Institute, Amsterdam, Netherlands; ^3^Department of Psychiatry, VU-University Medical Centre, Amsterdam, Netherlands; ^4^Department of Psychiatry, Academic Medical Center, Amsterdam, Netherlands; ^5^Department of Clinical Psychology, VU-University Medical Centre, Amsterdam, Netherlands

**Keywords:** psychoses, cardiovascular disease, standardised mortality ratios, smoking, metabolic syndrome, life expectancy

## Abstract

**Aims:** We aimed to identify baseline predictors of mortality in patients with a severe mental illness (SMI) over a 6-year period and to describe mortality rates as standardised mortality ratios (SMRs). We hypothesised that cardiovascular diseases, older age, cigarette smoking, more severe psychiatric symptoms and more severe psychotropic side effects, and alcohol or drug use were independent risk factors for mortality.

**Method:** Medical examinations were conducted at baseline in a cohort of 322 SMI patients. SMRs were estimated after 6 years and an evaluation was made of the impact of a wide range of variables on survival time.

**Results:** Almost 11% of the SMI patients had died at the end of the study period. All-cause SMRs were 4.51 (95% CI 3.07–5.95) for all SMI patients (4.89, 95% CI 2.97–6.80 for men, and 3.94, 95% CI 1.78–6.10 for women). Natural causes accounted for 86% of excess mortality and unnatural causes for 14%. Cardiovascular disease was a major contributor to this excess mortality. Multivariate Cox regression analyses showed that premature death was associated with a longer history of tobacco use (HR: 1.03, 95% CI 1.02–1.03) and more severe symptoms of disorganisation (HR: 2.36, 95% CI 2.21–2.52).

**Conclusions:** The high SMR and the incidence of cardiovascular disease-related death in SMI patients in our study justify concern. This study underscores the urgent need for interventions to reduce excess mortality in patients with SMI.

## Introduction

Researchers have consistently shown that mortality rates in patients with a severe mental illness (SMI) are excessively high. SMI patients die about 10–20 years earlier than the general population (1–5). Most systematic reviews showed that all-cause mortality in SMI patients is 2 to 3.5 times higher than in the general population (3–18). There is also evidence that this mortality gap has been increasing over time (4, 19, 6). This seems to indicate that SMI patients benefit less than the general population from progressions in healthcare (6). Reduced life expectancy in SMI patients is not only explained by unnatural causes of death like suicide and accidents but also by physical illnesses (8, 20–23). Studies have shown that cardiovascular diseases (CVD), respiratory and metabolic diseases are the main physical illnesses in patients with SMI, followed by cancer and infectious diseases (15, 20, 21, 24, 25).

The World Health Organisation has clustered different risk factors for excess mortality in SMI patients into three groups (26). Premature death in SMI is considered to be the result of 1) individual factors (disorder specific: e.g. genetic factors, severity of disorder, family history, early age of onset, reduced responsiveness to pain; behaviour-specific: e.g. poor life-style), 2) health system factors (e.g. deficiencies in leadership, financing, information and medications) and (3) social determinants of health (e.g. insufficient public policies, socio-economic position, environmental and social support vulnerabilities) (5, 27).

Although many studies have shown evidence of excess mortality in SMI patients, this has not led to a reduction of the mortality gap. In fact, underlying mechanisms are still not fully known and the data are fairly inconsistent (2). Several reviews showed for example that there is still considerable debate about the long-term effects of antipsychotic medication (27–30). There can be a delay of several years between drug-induced weight gain, the development of metabolic syndrome and premature death (27). Finally, there is a relative paucity of studies evaluating the link between symptom severity (in the mental disorder) and other baseline measures like psychotropic side effects and premature death. To our knowledge, only one study has found an association between disease characteristics like symptom severity and mortality (31), and only one study took place in the Netherlands. In this study we aim to contribute to the knowledge of risk factors and correlates of excess mortality in SMI patients. We hope that this allows us to develop better prevention programmes and interventions targeting predictors of early mortality.

This study is part of a larger study program (32–37). The objective of the present study was to examine premature mortality among SMI patients treated by mental healthcare institutions of Amsterdam (The Netherlands), and to calculate mortality ratios standardised to the general population. Baseline predictors of mortality in SMI patients were identified over a 6-year period and for purpose of exploration a prediction model was constructed. We hypothesised that cardiovascular diseases, older age, cigarette smoking, more severe psychiatric symptoms and more severe psychotropic side effects, and alcohol or drug use were independent risk factors for mortality. With previous literature lacking theoretical support for interaction between the above-mentioned predictors, no specific prediction model was *a priori* expected.

## Methods

### Design

This prospective longitudinal study was conducted between 2005 and 2011 in a survey sample of SMI patients treated by the mental healthcare institutions Arkin, Geestelijke Gezondheidszorg [GGZ (mental healthcare)] InGeest, Regionale Instelling voor Bescherm Wonen [(RIBW [Regional Institute for Sheltered Housing)] and Huis voor Onbehuisden [HVO (Sheltered housing for Homeless people)] Querido in Amsterdam, The Netherlands) (37), which are responsible for the treatment of SMI patients residing in Amsterdam. The main objectives were to evaluate changes in quality of life, disease characteristics, general functioning, care needs, social network, inclusion in society and victimisation (32–37). The current study describes the baseline predictors of mortality in SMI patients over a 6-year period. Baseline measurements started on August 2005. Data about sociodemographics, severity of psychopathology, substance use, use of antipsychotics, polypharmacy and side effects were obtained at baseline. The study was approved by the Dutch Association of Medical-Ethical Appraisal Committees (NVMETC) for mental health organisations.

### Population, Inclusion and Exclusion Criteria

For this research program we were specifically interested in a population of patients with functional impairment and chronic conditions, as treated by long-term transmural treatment teams [like (function) assertive community treatment teams] or patients treated in psychiatric hospitals, with a history of intensive mental healthcare during the previous 2 years. Patients were included when they fulfilled: 1) the criteria for a psychiatric diagnosis according to the Diagnostic and Statistical Manual of Mental Disorders IV (DSM-IV). 2) Where treated by long-term transmural treatment teams or treated in a psychiatric hospital. 3) Had a history of intensive mental healthcare during the previous 2 years. To define persistency of the psychiatric illness we used the criterion “a history of intensive mental healthcare during the previous 2 years”, which is based on SMI definitions by Delespaul et al. (38), Parabiaghi (39), Scheurs and Wiersma (40), Kroon et al. (41) and Ruggeri et al. (42). Further inclusion criteria for the target population were: adequate mastery of Dutch or English and residence in the Amsterdam district for at least 1 year. Exclusion criteria included being unable to understand questions or communicate, or an inability or unwillingness to give informed consent. Patients with comorbid substance use disorders were also included when they fulfilled the criteria listed here. Diagnoses were made by the attending psychiatrist (32, 33).

### Enrollment

Stratified random sampling was used, to select 876 patients from 2,846 patients treated in outpatient teams, sheltered housing facilities and inpatient care facilities. The aim was to include an equal number of patients from three care settings. After written informed consent was obtained, the face-to-face assessment was conducted by a trained psychologist, research assistants, or a senior researcher. The interviews took place in the patients’ own homes or, if preferred by the patients, at the healthcare centre. The interviews took approximately one and a half hours and patients received €15. Included patients were assessed again 6 years after inclusion (32, 33, 37).

#### Representativeness of the Sample

Representativeness analyses of our sample were done and published by Theunissen et al. (37). These analyses showed that included outpatients had more often a psychotic diagnosis, were slightly older, had a lower Global Assessment of Functioning (GAF), than patients who did not participate in the study. This may indicate that included outpatients were more severely ill than outpatients who did not participate in this study. There were no significant differences between involved inpatients and inpatients who did not participate in this study. In addition, inpatients were over-represented in our sample group (38%) by comparison with the general SMI population (13%) (37).

### Measures

Data about causes of death were obtained from electronic case files. Case notes were examined by a psychologist and a senior researcher to identify all causes of death between 01/08/2005 and 02/03/2012, and death certificates were obtained from the city +++records.

The Brief Psychiatric Rating Scale-Expanded or BPRS-E, a semi-structured interview, was used to measure psychiatric symptoms (43, 44). It consists of 24 items that can be scored on a seven-point Likert scale on the basis of observations during the interview and patient self-reports. The items are grouped in four subscales: positive symptoms, negative symptoms, depression and disorganisation (44). The psychometric properties of the BPRS-E are mostly good (45–47).

Measurements in the Addictions for Triage and Evaluation (MATE) was used to measure total number of years of smoking, current alcohol and drugs use, substance dependence and abuse. The MATE provides 20 different assessment scores and inter-rater/interviewer reliability is good (48).

Antipsychotic polypharmacy was defined as the use of more than one antipsychotic agent, which is a broadly used definition (13, 27, 28, 49–52).

Side effects of neuroleptics were assessed with The Liverpool University Neuroleptic Side Effect Rating Scale (LUNSERS). Psychological, neurological, autonomic, hormonal and other miscellaneous side effects were assessed using 41 items. Ten (‘red herring’) items were used to detect response tendencies. Patients described side effects on a five-point scale ranging from ‘not at all’ to ‘very much’ side effects, with a higher score indicating more severe side effects. The test-retest reliability of the LUNSERS is good (*r* = .811, *P* < .001), as is concurrent validity by comparison with the ‘Udvalg for Kliniske Undersøgelser’ (UKU, task force for clinical investigations) Side Effect Rating Scale for Neuroleptics (*r* = .828, *P* < .001) (53).

### Data Analysis

Frequency distributions were used to describe causes of death and the corresponding ICD-10 codes (54).

Standardised mortality ratios (SMRs) were used to describe the relative risk for SMI patients and the general population. SMRs were calculated for the study period using the number of deaths observed divided by the expected number of deaths, controlling for age and gender. The expected number of deaths came from gender-specific mortality statistics for the Dutch population between 2005 and 2011^1^. The change in gender-specific expected number of deaths were estimated for each year that an individual became older during follow-up. Because not all ages were equally prevalent, we weighted by age. An SMR greater than 1.0 indicates that the relative risk of death for SMI patients is higher than that of the general population (10).

The defined daily dose (DDD) system of the *World Health Organization* (WHO) Collaborating Centre for Drug Statistics Methodology was used as an equivalent to compare prescriptions of antipsychotics at baseline. *The DDD is the assumed average maintenance dose per day for a drug used for its main indication in adults (WHO)*. It is the most frequently used system in academic articles and reports, and it is a reliable tool for the standardisation of drug doses (55). The following WHO formula was used to calculate the DDD for each antipsychotic:

*Drug usage (DDDs) = (items issued x amount of drug per item)/DDD*. DDDs greater than 1 indicate a dosage that is higher than average. DDDs greater than 1.5 were classified as excessive (56–58).

Univariate Cox proportional hazard regression analysis was used to assess associations between baseline variables and mortality (REF). The following variables were used as baseline measures: age, gender, ethnicity, diagnosis, symptom severity, substance use, antipsychotic treatment and dose, polypharmacy and side effects.

Cox proportional hazard multivariate regression analysis was used to assess the effects of covariates (backward method) on mortality rates (59). Covariates were: symptoms of disorganisation and duration of cigarette smoking. Time to death was used as the time variable. The presence of anticholinergic side effects was omitted as a covariate because of the violation of the proportional hazards assumption. We considered ethnicity as potential confounders in the multivariate Cox proportional hazard regression (enter method). To avoid multicollinearity between the predictors age and duration of cigarette smoking, we omitted age as a predictor. To examine if there was relevant confounding a change of more than 10% between regression coefficients was used (59).

Sensitivity analyses were performed to examine if predictors were influenced by heterogeneity in diagnoses. All analyses belonging to **Table 2** were re-done, and only applied to patients with a psychotic disorder with high levels of psychiatric burden (The Global Assessment of Functioning (GAF) scale ≤ 50) (41). However, number of observations were too limited to apply a sensitivity analysis to the multivariate Cox regression model.

All analyses were performed with SPSS 22 (SPSS Inc., 2009), and statistical significance was defined as *P < 0.05*.

## Results

In 2006, 876 patients were selected. Some patients refused to participate (25.9%) and others did not participate for unknown reasons (25.5%). Another 2.9% of the patients were not included because their clinicians deemed that inclusion would have a negative impact on clinical status. Patients who were no longer receiving treatment (8.3%) were also excluded. Another patient (0.1%) was excluded because no diagnosis was present. Data for five patients (0.6%) were missing: one removed his patient file and four left the country. These five patients were excluded because their survival status could not be verified. The remaining 322 (36.8%) patients were included in the study.

The mean age of the included patients was 54.9 at baseline. Approximately 80% of the patients had a psychotic disorder; 21% had non-psychotic disorders: often a severe mood (46.3%), substance use (50.7%) or personality disorder (3%). Almost 80% of the patients were using antipsychotic medication (SD = 0.34; range, 1–5) at baseline.

During the follow-up period of 6 years, 35 patients died (10.9%: men 11.6%; women 9.8%). The mean age of death for all these patients was 59.6 (SD = 12.19; range, 32–88), 59 for men (SD = 10.85; range, 32–78) and 60 for women (SD = 14.89; range, 41–88). The overall SMR for the cohort was 4.51 (95% CI 3.07–5.95 for all SMI patients; 4.89, 95% CI 2.97–6.80 for men, and 3.94, 95% CI 1.78–6.10 for women). **Table 1** lists the causes of death.

**Table 1:** Cause of death by age, gender and location (N = 35).

	ICD-10	Total		Age	Gender				Location^a^			
					Male		Female		Own home	Shelter	Psychiatric hospital	Hospital
		n	%	Mean	n	%	n	%	n	n	n	n
Total	35		59.6	23		12		5	10	15	5	
Natural cause of death												
Diseases of the circulatory system	I00–I99	11	31.4	53.8	6	26.1	5	41.7	1	3	7	0
Diseases of the digestive system	K00–K93	5	14.3	61.2	4	17.4	1	8.3	0	4	1	0
Diseases of the nervous system	G00–G99	1	2.9	62.4	1	4.3	0	0	0	1	0	0
Diseases of the respiratory system	J00–J99	2	5.7	69.7	2	8.7	0	0	0	1	0	1
Neoplasms	C00-D48	2	5.7	52.9	2	8.7	0	0	0	0	1	1
Certain infectious and parasitic diseases	A00-B99	2	5.7	52.0	1	4.3	1	8.3	1	0	0	1
Diseases of the genitourinary system	N00–N77	1	2.9	66.7	1	4.3	0	0	0	0	1	0
Endocrine, nutritional and metabolic diseases	E00–E90	1	2.9	39.5	0	0	1	8.3	0	0	0	1
Undetermined	E980–E989	5	14.3	65.6	3	13.0	2	16.7	1	0	3	1
Unnatural causes of death												
External causes of morbidity and mortality	V01-Y98	2	5.7	38.8	2	8.7	0	0	0	0	2	0
Euthanasia^b^		3	8.6	49.2	1	4.3	2	16.7	2	1	0	0

a Last known health location.

b Euthanasia in terminal illnesses.

Death from natural causes accounted for 86% of deaths. More details about natural causes found on five occasions or more can be found below. Death from diseases of the circulatory system accounted for 31% of mortality, with most of these patients dying from ischaemic heart disease or acute myocardial infarction in a psychiatric hospital. Fifty percent of the patients who died at the age of 50 or lower died from diseases of the circulatory system. Five patients (14.3%) died from diseases of the digestive system. Five patients (14.3%) died from unknown causes.

Death resulted from unnatural causes in five patients (14.3%). Almost 6% committed suicide and approximately 9% dies as a result of euthanasia.

**Table 2** lists the results of the univariate analysis of associations between mortality and sociodemographics, clinical characteristics, substance use and medication at baseline. Mortality was elevated in older subjects, those with western ethnicity, more symptoms of disorganisation, a longer duration of smoking and with more severe anticholinergic side effects at baseline. A trend was found for an association between non-psychotic disorders and mortality. Mortality seemed higher in males but this association was not statistically significant. The prevalence of cigarette smoking was almost 70%. Fourteen percent smoked 10 years or less, approximately 35% smoked 11–30 years, and 20% smoked for more than 31 years. Sensitivity analyses revealed that in patients with a psychotic disorder, same predictors were found, with the exception of anticholinergic side effects.

**Table 2:** Univariate Cox regression analysis of the associations between mortality and sociodemographics, clinical characteristics, substance use, medication and side effects (*N* = 322).

	Still alive (*n* = 287)		Observed deaths (n = 35)		Hazard ratio	95.0% C.I.		*P* ^a^
	Mean (*n)*	SD (%)	Mean (*n*)	SD (%)				
**Sociodemographics**								
Age at baseline	45.1	10.5	56.2	11.6	1.086	1.055	1.118	0.000
Gender								
Male	(176)	(61.3)	(23)	(65.7)	1.215	0.604	2.441	0.585
Female	(111)	(38.7)	(12)	(34.3)				
Ethnicity^b^								
Western	(188)	(66.9)	(32)	(91.4)	4.967	1.521	16.221	0.008
Non-Western	(93)	(33.1)	(3)	(8.6)				
**Diagnosis**								
Psychotic disorder	(231)	(80.5)	(24)	(68.6)	1.843	0.902	3.762	0.093
Non-psychotic disorder	(56)	(19.5)	(11)	(31.4)				
Dual diagnosis								
Yes	(79)	(27.5)	(5)	(14.3)	2.171	0.843	5.597	0.108
No	(208)	(72.5)	(30)	(85.7)				
**Symptoms (BPRS-E)** **^b^**								
Positive symptoms	1.7	0.9	1.9	1.0	1.146	0.742	1.770	0.539
Negative symptoms	1.4	0.6	1.6	0.7	1.580	0.939	2.661	0.085
Depression and anxiety	1.9	0.8	1.9	0.8	1.067	0.639	1.780	0.805
Disorganisation^d^	1.4	0.5	1.6	0.7	2.073	1.145	3.751	0.016
BPRS total	1.6	0.5	1.8	0.6	1.777	0.860	3.668	0.120
**Substance use (MATE)** **^c^^,^^d^**								
Alcohol	5.7	10.5	8.7	12.8	1.019	0.993	1.047	0.153
Cannabis	3.5	8.9	2.1	6.4	0.979	0.932	1.027	0.382
Hard drugs^e^	2.3	8.3	3.4	9.2	1.011	0.979	1.044	0.493
Duration of cigarette smoking, years	15.8	14.2	26.3	20.0	1.043	1.021	1.066	0.000
**Antipsychotic drug dose (PDD/DDD) at baseline** **^f^**								
Normal or lower than the average dose	(110)	(38.3)	(14)	(40.0)	1.140	0.478	2.717	0.768
Higher than the average dose	(32)	(11.1)	(4)	(11.4)	1.135	0.342	3.769	0.836
Excessive dose	(75)	(26.1)	(8)	(22.9)				
No antipsychotic medication or missing dose^g^	70	(24.4)	9	(24.4)				
**Polypharmacy at baseline** **^h^**								
Number of antipsychotics^i^	1.1	0.6	1.1	(0.6)	1.038	0.583	1.847	0.899
Number of somatic and antipsychotic medication	3.1	1.8	3.7)	(2.4)	1.165	0.972	1.397	0.098
**Side effects (LUNSERS)** **^j^**								
Extrapyramidal side effects	4.5	4.3	5.0	4.9	1.026	0.954	1.104	0.484
Psychic side effects	8.9	7.4	9.2	8.4	1.004	0.959	1.052	0.858
Anticholinergic side effects	3.0	3.5	4.4	4.4	1.087	1.002	1.179	0.045
Allergic side effects	1.5	2.3	1.9	2.3	1.055	0.920	1.211	0.442
Other autonomic side effects	2.8	3.6	3.3	4.2	1.031	0.944	1.126	0.493
Hormonal side effects	2.1	3.3	1.9	3.43	0.983	0.880	1.098	0.764
Miscellaneous	2.4	2.5	2.4	2.6	1.002	0.874	1.149	0.976

a P is the result of a Cox regression. Significant findings shown in bold.

b BPRS-E, Brief Psychiatric Rating Scale—Expanded.

c MATE, Measurements in the Addictions for Triage and Evaluation.

d Number of days of substance use in the last month.

e Cocaine, stimulants, 3.4-methylenedioxy-methamphetamine, opiates.

f PDD, prescribed daily dose; DDD, defined daily dose.

g Excluded from Cox regression.

h Polypharmacy was defined as the concomitant use of two or more antipsychotics.

i Controlled for dosing.

j LUNSERS, the Liverpool University Neuroleptic Side Effect Rating

To further our understanding, we performed a Cox regression (backward method), entering the significant predictors from **Table 2** as covariates. In the analysis we adjusted for ethnicity. **Table 3** lists the results of the Cox regression model. We found the mortality risk of patients with SMI increases by a factor of 1.036 (0.36%) for each year that a patient has smoked. Finally, the mortality hazard increases with a factor of 2.310 (131%) for each one-point increase in the disorganisation score.

**Table 3:** Multivariate Cox regression predicting likelihood of reduced survival in patients with SMI (N = 322).

	Hazard ratio	95.0% C.I.^a^		P^b^
Ethnicity				
Western	2.658	0.782	9.038	0.117
Non-Western	*Reference*			
Duration of smoking, year	1.036	1.007	1.066	0.014
Disorganisation	2.310	1.232	4.332	0.009

Adjusted for ethnicity.

a Confidence Interval for hazard ratio.

b Omnibus test, model P = 0.002; −2 log likelihood: 223.372 8.

## Discussion

This study confirms that SMI patients have an increased risk of premature mortality. We found a standardised mortality rate after 6 years of follow-up that was four to five times higher than in the general population in the Netherlands. In our random sample, almost 11% of the SMI patients died in a period of 6 years, in particular from cardiovascular disease. Death from natural causes accounted for 86% of the excess mortality and unnatural causes for 14%. The highest mortality rates were found in patients receiving residential treatment in psychiatric hospitals. Reduced survival was predicted by: higher age, Western background, more years smoking cigarettes, symptoms of disorganisation and anticholinergic side effects of antipsychotics. In multivariate analyses, number of years of cigarette smoking, and symptoms of disorganisation remained as predictors of a higher risk of premature death.

Total SMR (4.51) in our cohort was higher than in other studies (4.51, as compared to 1.2–4.9) (3, 6, 8–10, 14, 15, 18, 60), with most studies reporting an SMR of 2.5 (3). Most patients died of natural causes, including diseases of the circulatory system (cardiovascular-related), a finding that was in accordance with our expectations and the results of other studies, (2, 7, 10, 15, 20, 61). Moreover, this finding is also in line with the common causes of death in the general population^2^. We found lower rates of cancer than Brown et al (7).

Most patients died in psychiatric hospitals. This is not surprising given the poorer medical and psychiatric condition of these patients by comparison with outpatients (11). In line with prior research (15, 62–65) with a follow-up range from 4 to 10 years, we found a suicide rate of 0.6%. The distribution of natural (86% *vs* 60–67.3%) and unnatural (14% *vs* 17.5–40%) deaths in our study is only slightly different from that in other studies (4, 20–22). In our study, three patients underwent euthanasia. It is worth noting that the Netherlands was the first country in the world where euthanasia was legal for patients suffering unbearable pain with no prospects of recovery. Euthanasia has now also been legalised in Belgium, Luxembourg, Colombia and Canada (66), several states of the United States and Mexico. As a result, data about euthanasia are very scarce and comparisons are therefore impossible. It is known that fatal accidents are more prevalent in SMI patients than in the general population (2, 10, 67). By contrast with previous studies, none of our patients died due to an accident (2, 8, 68).

In line with our expectations relating to severe psychiatric symptoms, we identified disorganisation as a predictor of premature death. To our knowledge, no other study has found this specific association. Previous studies have found that more positive symptoms (31) and fewer negative symptoms (69) were associated with higher mortality rates in SMI patients. One potential explanation for the higher risk of premature mortality in disorganised patients is that these patients may have the most severe psychiatric problems, possibly impairing their ability to live healthily and to seek somatic care when needed.

The finding that there was no difference in the association between antipsychotic monotherapy or polypharmacy and mortality is consistent with studies from Tiihonen et al. (27), Tenback et al. (24) and Baandrup et al. (70), but it contradicts research from Auquier et al. (20), Joukamaa et al. (13) and Waddington et al. (52), who found that polypharmacy was associated with a higher mortality risk. More specifically, we also found no association between DDDs and mortality. Other studies have found that higher prescribed doses of antipsychotics are linked to a higher mortality rate (31) and to an increased risk of mortality from coronary heart disease and stroke (61).

Unfortunately, the heterogeneity of the designs of these other studies preclude direct comparison. Furthermore, most of the studies mentioned here looked at drug prescriptions throughout the study period although, like us, Joukemaa et al. (13) and Loas et al. (31) measured drug prescriptions at baseline.

A high mortality risk was also seen in patients with more severe anticholinergic side effects. To our knowledge, no other study has found this specific association. However, the interpretation of this finding, in which only this type of psychotropic side effect is associated with early death, is challenging and, in addition, future research should test this finding further before proceeding to interpret it. Contrary to our hypothesis, we found no association between substance use and mortality. This could be explained by the fact that substance use was very low in our SMI group. Despite low hazard ratios, our findings support the evidence that cigarette smoking is a risk factor for premature mortality (3, 6, 8, 18, 71). The finding that Western ethnicity was associated with reduced life expectancy is in accordance with a study by Olfson et al. (15). Further research is needed to clarify the mechanism underlying this finding.

### Limitations

This study has several limitations. Firstly, we have to address the issue of survivor treatment selection bias, also known as immortal time bias (72, 73). Which was for example correctly addressed by Tiihoonen et al. (27); Cullen et al. (74); en Tornianen et al. (75), while others did not applied sensitivity analyses to address immortal time bias. In our study, baseline measurements were at one point in time. Patients who did not use any medication but started for example a few days after baseline were classified as the non-users group. This may have resulted in misinterpretations of the classifications. Also, patients who moved to foreign countries and therefore dropped out of treatment, may have died. From these patients we could not check the death registers. Although this happened not very often, this may have led to slightly underestimated death rates. It is also unclear if polypharmacy is related to an increased mortality risk, due to a direct toxic effect. Or that here may be a consequence of a cohort effect: patients with polypharmacy may be psychiatrically and physically sicker than patients with monotherapy. Thus when patients with and without polypharmacy were compared, underlying individual characteristics may differ. Baseline measurements only were used to predict mortality. We were therefore unable to take changes in medication or dosage, or the discontinuation of pharmaceutical treatment, into consideration, even though these are not uncommon (76). The total duration and cumulative antipsychotic dosage may therefore be a better measure to use in studies of reduced survival in SMI patients (61, 77). Moreover, we did not corrected for changes from one antipsychotic to another. Some patients might need to decrease one antipsychotic gradually, whilst raising another antipsychotic agent. Despite the fact that we do not think that this was highly prevalent, it is possible that the polypharmacy rate is slightly overestimated. Therefore the results of antipsychotics exposures among people with SMI has to be interpreted carefully. Secondly, our sample consisted of people with SMI with a mean age of 54.9 years at baseline and many of these people will have been ill for many years. Some patients died before it was possible to include them in the present study. This means that there may have been oversampling of healthy people. However, in clinical practice, we still see people of this age with a long history of SMI. Furthermore, we are interested in the risks to which this age group is exposed. A better understanding of these risk factors will help clinicians to address these the risk factors and identify potential solutions.

Thirdly, this is an observational study and so it was not possible to control for all potential confounding variables. Important predictors of mortality rates such as body weight and lifestyle factors such as physical exercise and diet may exist (30), but, unfortunately, they were not assessed in this study.

Fourthly, we did not control for confounding from indication in relation to polypharmacy, DDD or somatic medication, because the specific reason for the prescribed antipsychotic (combination) treatment was not recorded. Antipsychotics are also sometimes (off-label) prescribed for patients with without a psychotic disorder (78). We assumed that the psychiatrist made an informed decision and that medication prescriptions were tailored to each patient’s individual need. Therefore, we did not checked for contraindication. Future research should address this point.

Fifthly, another issue is that we included only patients who were receiving psychiatric care and this may have led to an underestimate of mortality in patients with SMI. Patients with SMI without psychiatric care probably live in poorer circumstances and may have an even higher SMR. Our results may therefore not be representative for the general SMI population and SMR ratios may have slightly been underrated. Another issue is the representativeness of our sample. It seems that our subgroup of outpatients are a more severe group and that inpatients were overrepresented. The representativity of the subgroups of inpatients and patients living in a sheltered care facility was good. In contradiction to our limitation what we mentioned before, this may have resulted in overrated SMR ratios for patients who receive psychiatric care.

Moreover, conclusions were based on small numbers, with limited statistical power. Therefore, results has to interpreted carefully. Finally, it was impossible to perform reliable statistical analyses to identify the determinants of specific diseases since the numbers in the different ICD-10 categories were too low.

## Conclusions and Implications

The high SMR and the incidence of CVD-related deaths in SMI patients in our study justify concern, particularly given the apparent potential for improvement given the fact that SMI patients with cardiovascular disease and diabetes use healthcare less than other diagnostic groups and others in the community with the same level of physical health problems (79). It is also alarming that most excess mortality is associated with preventable conditions (21, 80, 77). Our findings therefore imply that improvements in care are necessary to reduce excess mortality in SMI patients. The evidence suggests that the following modifiable risk factors should be covered by an intervention to reduce excess mortality in SMI: metabolic risk factors (abdominal obesity, elevated blood pressure, dyslipidaemia and insulin resistance, endothelial dysfunction, inflammatory activity and stress), lifestyle risk factors (cigarette smoking, substance use, physical inactivity and poor diet) and improved access to medical health services (3, 25, 71, 80, 81).

Our findings support the idea that we should improve early medical screenings to identify acute medical disease and lifestyle interventions focused on reducing the risk of premature death (77). In the light of the high mortality rates in our study, we started to provide counselling through an online intervention: www.Leefstijlinbeeld.nl. This interactive tool allows patients to assess their lifestyle, either under the supervision of their counsellor or on their own. It provides patients with direct feedback, for example about smoking, nutritional intake and other lifestyle factors, and suggestions for improvement. However, the long-term effects of lifestyle interventions are rather poor and future research should explore the effectiveness of modified interventions. Finally, the excess mortality we found highlights the importance of an increased focus from professionals on the reduction of modifiable risk factors and the establishment of a policy for integrating mental and physical healthcare in order to prevent avoidable deaths.

## Author's Note

We are aware that data collection for this paper has been submitted eight years after completion of data collection. The reason for this is that we already published five papers in international peer reviewed journals, submitted another two papers, and wrote two comprehensive reports between data collection and submission of this paper.

## Data Availability Statement

The datasets generated for this study are available on request to the corresponding author.

## Ethics Statement

The study was approved by the Dutch Association of Medical-Ethical Appraisal 91 Committees (NVMETC) for mental health organisations. The patients/participants provided their written informed consent to participate in this study.

## Author Contributions

Conceived the study: LM, MK, JT, LH, AB, PD, HV, JD. Designed the assessment: MK, JD, JT. Carried out the data acquisition: LM, PD. Drafted the manuscript: LM. All authors read and approved the final manuscript.

## Funding

This study was supported by the Stichting tot Steun VCVGZ grants ST13102.ME2 (Principal investigator, PhD JD).

## Conflict of Interest

The authors declare that the research was conducted in the absence of any commercial or financial relationships that could be construed as a potential conflict of interest.

## Footnotes

1 CBS. Statline. Retrieved from http://tinyurl.com/CBS-Statline-Lifespan

2 CBS. (2013). CBS Satline Causes of death. Retrieved from http://tinyurl.com/CBS-Cancer-CVD
